# The genome sequence of black poplar, *Populus nigra* *subsp.* *betulifolia *L., 1753 (Salicaceae)

**DOI:** 10.12688/wellcomeopenres.21300.1

**Published:** 2024-04-24

**Authors:** Maarten J. M. Christenhusz, Heloise Bastiaanse, Royal Botanic Gardens Kew Genome Acquisition Lab, Plant Genome Sizing collective, Darwin Tree of Life Barcoding collective, Wellcome Sanger Institute Tree of Life Management, Samples and Laboratory team, Wellcome Sanger Institute Scientific Operations: Sequencing Operations, Wellcome Sanger Institute Tree of Life Core Informatics team, Tree of Life Core Informatics collective, Darwin Tree of Life Consortium

**Affiliations:** 1 Royal Botanic Gardens Kew, Richmond, England, UK; 2 Curtin University, Perth, Western Australia, Australia; 3 Department of Plant Biotechnology and Bioinformatics, Ghent University, Ghent, Belgium

**Keywords:** Populus nigra, black poplar, genome sequence, chromosomal, Malpighiales

## Abstract

We present a genome assembly from an individual *Populus nigra* subsp. *betulifola* (black poplar; Tracheophyta; Malpighiales; Salicaceae). The genome sequence is 413.2 megabases in span. Most of the assembly (99.73%) is scaffolded into 19 chromosomal pseudomolecules. Mitochondrial and plastid genomes were also assembled. Three mitochondrial assemblies have lengths of 281.85, 335.57 and 186.15 kilobases, and the plastid genome has a length of 156.37 kilobases.

## Species taxonomy

Eukaryota; Viridiplantae; Streptophyta; Streptophytina; Embryophyta; Tracheophyta; Euphyllophyta; Spermatophyta; Magnoliopsida; Mesangiospermae; eudicotyledons; Gunneridae; Pentapetalae; rosids; fabids; Malpighiales; Salicaceae; Saliceae; *Populus*; *Populus nigra* L., 1753 (NCBI:txid3691) subsp. *betulifolia* (Pursh) Wettst., 1952.

## Background

The black poplar, *Populus nigra* L. is a large deciduous tree with a widespread distribution across Europe, northern Africa and east to central Asia ( POWO, 2024; Stanton *et al.*, 2010). This resilient species thrives in riparian ecosystems. It shows a remarkable tolerance to high water levels and sediment movements, and is often among the first species to colonise bare moist soil of riverbanks, making it vital to the establishment of riparian forests along river margins and in floodplains ( Imbert & Lefèvre, 2003; Lefèvre *et al.*, 1998). Such forests are not only biodiversity hotspots, but are also essential for the natural control of flooding, the prevention of bank erosion and the maintenance of good water quality ( Van Looy *et al.*, 2013).

Regrettably, the once widespread native *P. nigra* is now one of the most endangered tree species in Europe, mainly because of the loss of its natural alluvial habitat and due to competition and introgression with exotic poplar species ( Cagelli & Lefevre, 1995; Fossati *et al.*, 2004; Vanden Broeck *et al.*, 2005). For these reasons, *P. nigra* merits particular attention with regard to conservation efforts. In Britain, the wild-growing black poplar (i.e. *P. nigra* subsp. *betulifera*) is one of the rarest tree species, with only about 7000 individuals. In contrast, cultivars of the Black poplar are among the most cultivated trees in temperate latitudes, sometimes as the pure species, but often as interspecific hybrids (e.g. *Populus* x *canadensis* Moench., which is a cross with the American species *P. deltoides* (Eastern Cottonwood)). Such hybrids are renowned for their rapid growth and high biomass yield, as well as their effective coppice regeneration and vegetative propagation, making it a suitable crop for the production of a great variety of products including wood, biofuels, and pulp and paper ( Balatinecz & Kretschmann, 2001; Dickmann, 2006). Owing to its quality as a pioneer species, black poplar also provides a range of ecosystem services, including phytoremediation in polluted industrial zones, erosion control in river systems, and carbon sequestration by reforestation of lowlands in temperate regions ( De Rigo *et al.*, 2016; Di Baccio *et al.*, 2003; Zalesny *et al.*, 2016). Besides its economic and ecological importance, the poplar (including *P. nigra*) has become an important model tree genus for tree biotechnology ( Douglas, 2017; Ellis *et al.*, 2010). In part, this is due to its relatively small genome, and the ease with which it can be transformed.

Across its range, *P. nigra* displays remarkable phenotypic variation, but spontaneous hybridization makes taxonomic classification complex ( Cagelli & Lefevre, 1995). Three subspecies are currently accepted ( POWO, 2024), with *P. nigra* subsp. *caudina* (Ten.) Bugała found across Mediterranean Europe, *P. nigra* subsp. *betulifolia* found in northwestern Europe (Ireland, Britain, western France) and *P. nigra* subsp. *nigra* found across its range, but most common in central and eastern Europe, and Central Asia. Numerous forms and cultivars are known and many are easily vegetatively propagated, extensively planted as avenue trees and as wind breaks across Eurasia and beyond. The species is naturalised in North America, Argentina, South Africa, the Himalayas, East Asia and eastern Australia ( POWO, 2024). One of the most recognisable forms is the fastigiated Lombardy poplar, *P. nigra* cv. Italica. This cultivar was selected in Lombardy, northern Italy in the seventeenth century, after which is soon became popular as a parkland tree across Europe. Crosses of this cultivar with subsp. *betulifolia* (Plantières Group) are the main trees called Lombardy poplar in Britain and Ireland, as these are better suited to the cooler climate.

Here we present the first genome from a wild stand of *Populus nigra* subsp. *betulifolia*. We hope this resource will contribute to playing a pivotal role in the fields of functional genomics, genetic engineering and molecular breeding of this economically significant genus. The reference genome will enable the systematic assessment and characterization of sequence variation among natural populations of black poplar, without the need to rely on reference-guided mapping and variant calling based solely on the reference genome of *P. trichocarpa* Torr. & A.Gray ex. Hook. This will encompass not only Single Nucleotide Polymorphisms (SNPs), but also structural variants, known to influence the regulation of complex quantitative traits in poplars ( Bastiaanse *et al.*, 2019).

The genome will be particularly valuable in large system genetics analysis such as the ongoing ERC project POPMET (DOI10.3030/834923), a project that aims to integrate genomic, transcriptomic and metabolomic data for the identification of secondary metabolites, metabolic pathways and their genes in *P. nigra.* Finally, the availability of a *P. nigra* reference genome, combined with the existing suite of published *Populus* reference genomes, will empower precise assessments of synteny, recombination and chromosomal origins. This invaluable resource opens up exciting opportunities for the study of adaptive evolution in long-lived woody species.

## Genome sequence report

**Figure 1.**
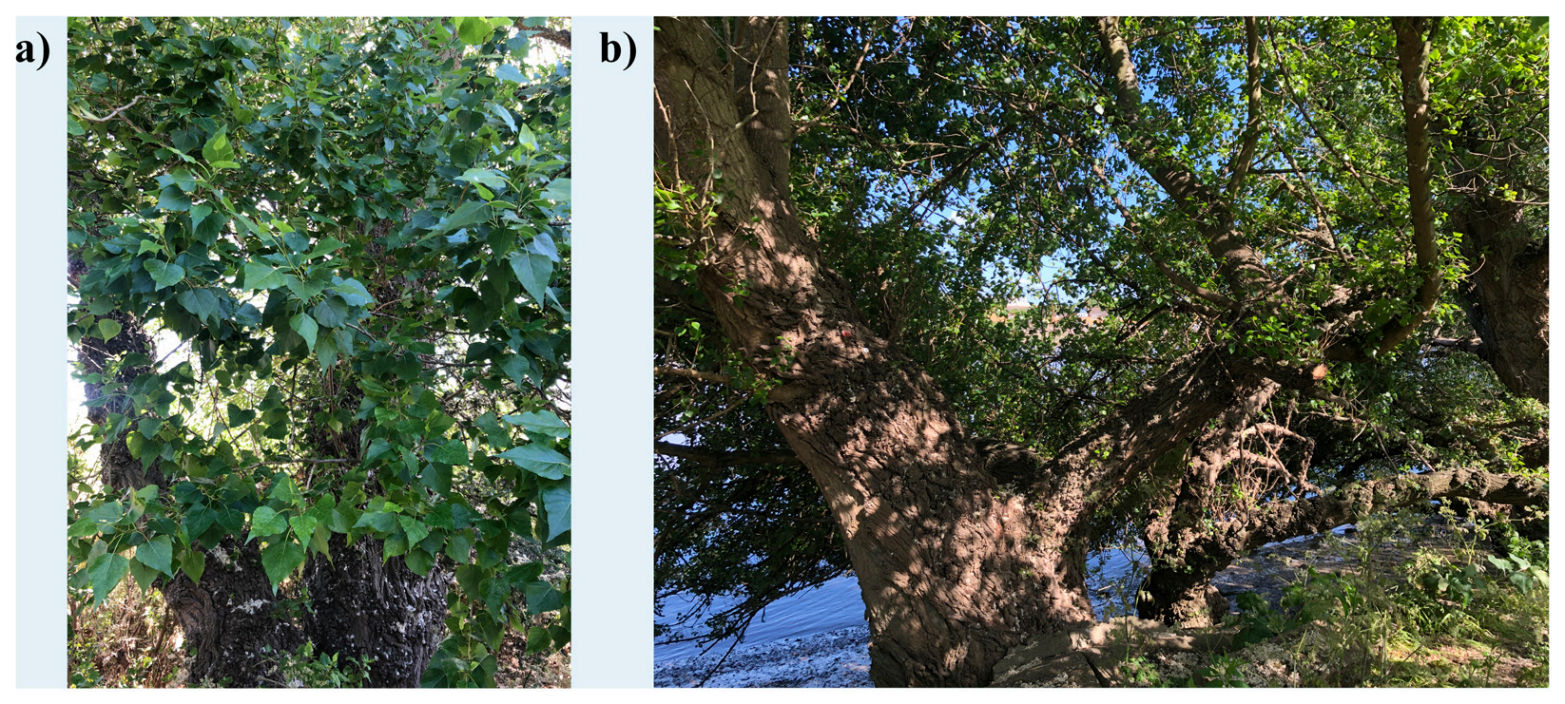
Photographs of the *Populus nigra* subsp. *betulifolia* (ddPopNigr1) specimen used for genome sequencing.

The genome was sequenced from a female individual of *Populus nigra* subsp *. betulifolia* ( Figure 1) collected from along the Thames towpath in Barnes, Richmond, Surrey, UK (51.48, –0.23). Using flow cytometry, the genome size (1C-value) was estimated to be 0.52 pg, equivalent to 500 Mb. A total of 54-fold coverage in Pacific Biosciences single-molecule HiFi long reads was generated. Primary assembly contigs were scaffolded with chromosome conformation Hi-C data. Manual assembly curation corrected 156 missing joins or mis-joins and removed 11 haplotypic duplications, reducing the assembly length by 1.66% and the scaffold number by 85.12%, and increasing the scaffold N50 by 25.44%.

**Figure 2.**
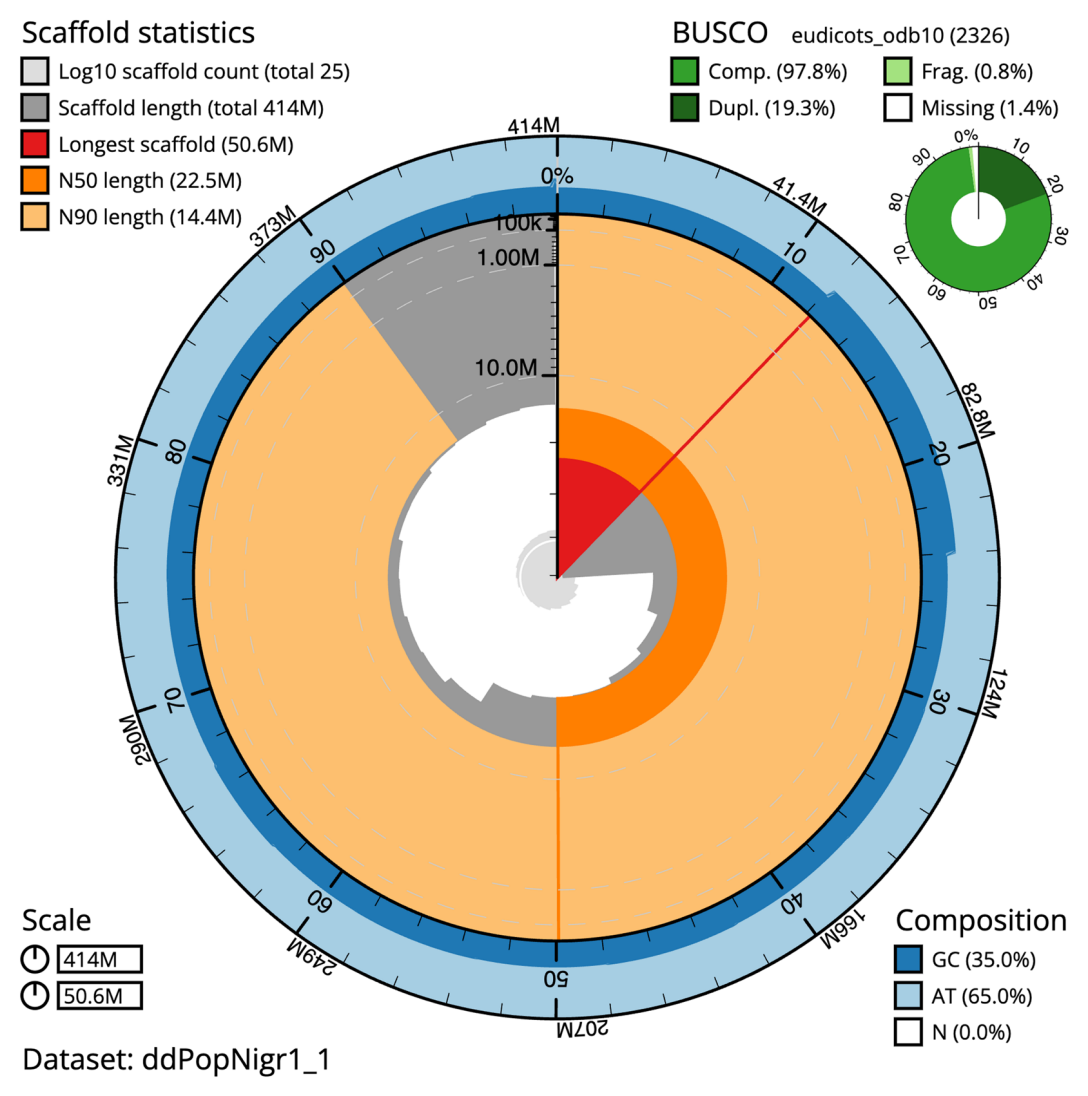
Genome assembly of *Populus nigra* subsp *. betulifolia*, ddPopNigr1.1: metrics. The BlobToolKit snail plot shows N50 metrics and BUSCO gene completeness. The main plot is divided into 1,000 size-ordered bins around the circumference with each bin representing 0.1% of the 414,184,750 bp assembly. The distribution of scaffold lengths is shown in dark grey with the plot radius scaled to the longest scaffold present in the assembly (50,570,738 bp, shown in red). Orange and pale-orange arcs show the N50 and N90 scaffold lengths (22,490,379 and 14,411,003 bp), respectively. The pale grey spiral shows the cumulative scaffold count on a log scale with white scale lines showing successive orders of magnitude. The blue and pale-blue area around the outside of the plot shows the distribution of GC, AT and N percentages in the same bins as the inner plot. A summary of complete, fragmented, duplicated and missing BUSCO genes in the eudicots_odb10 set is shown in the top right. An interactive version of this figure is available at https://blobtoolkit.genomehubs.org/view/ddPopNigr1_1/dataset/ddPopNigr1_1/snail.

**Figure 3.**
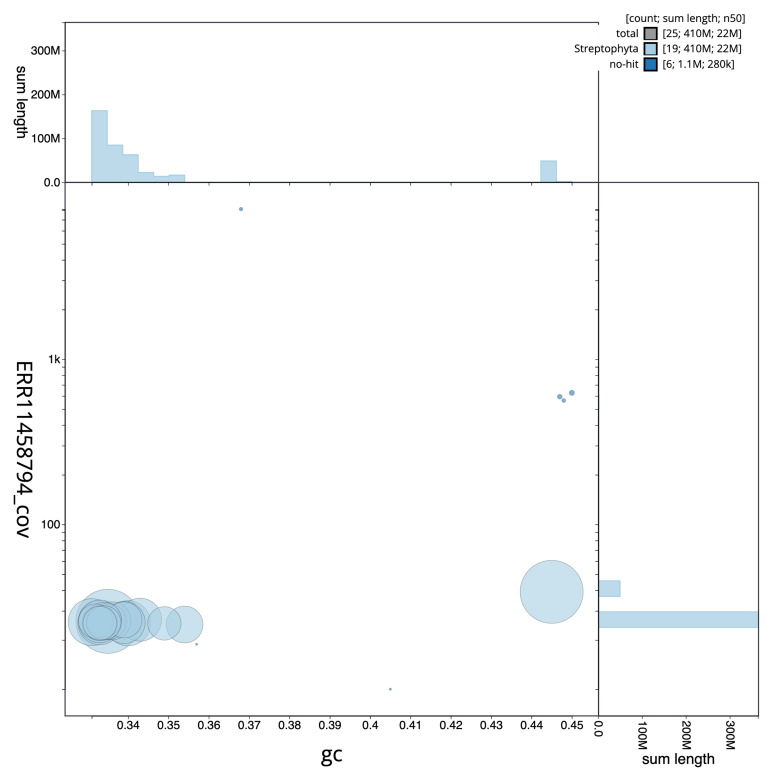
Genome assembly of *Populus nigra* subsp. *betulifolia*, ddPopNigr1.1: BlobToolKit GC-coverage plot. Scaffolds are coloured by phylum. Circles are sized in proportion to scaffold length. Histograms show the distribution of scaffold length sum along each axis. An interactive version of this figure is available at https://blobtoolkit.genomehubs.org/view/ddPopNigr1_1/dataset/ddPopNigr1_1/blob.

**Figure 4.**
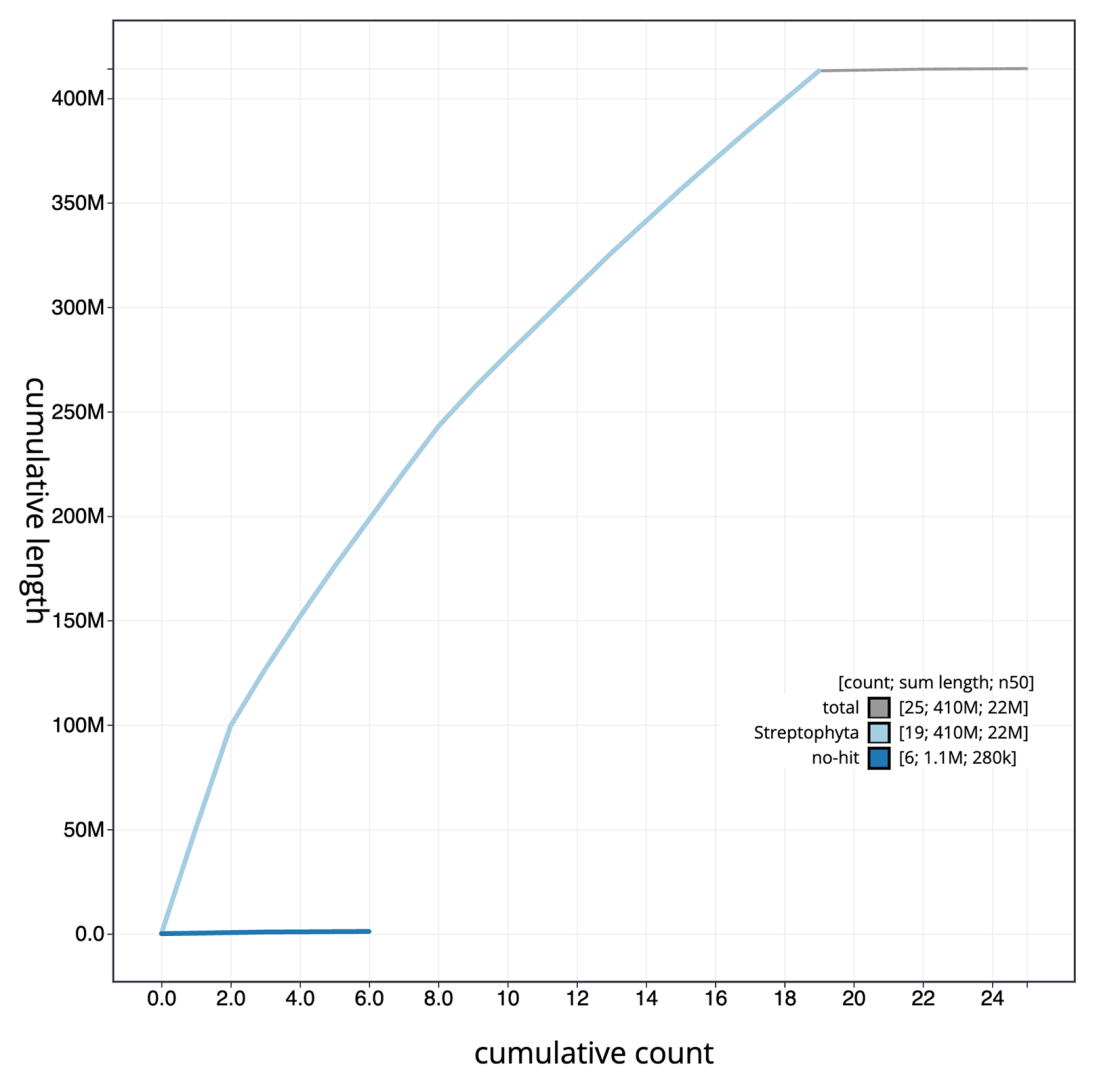
Genome assembly of *Populus nigra* subsp. *betulifolia*, ddPopNigr1.1: BlobToolKit cumulative sequence plot. The grey line shows cumulative length for all scaffolds. Coloured lines show cumulative lengths of scaffolds assigned to each phylum using the buscogenes taxrule. An interactive version of this figure is available at https://blobtoolkit.genomehubs.org/view/ddPopNigr1_1/dataset/ddPopNigr1_1/cumulative.

**Figure 5.**
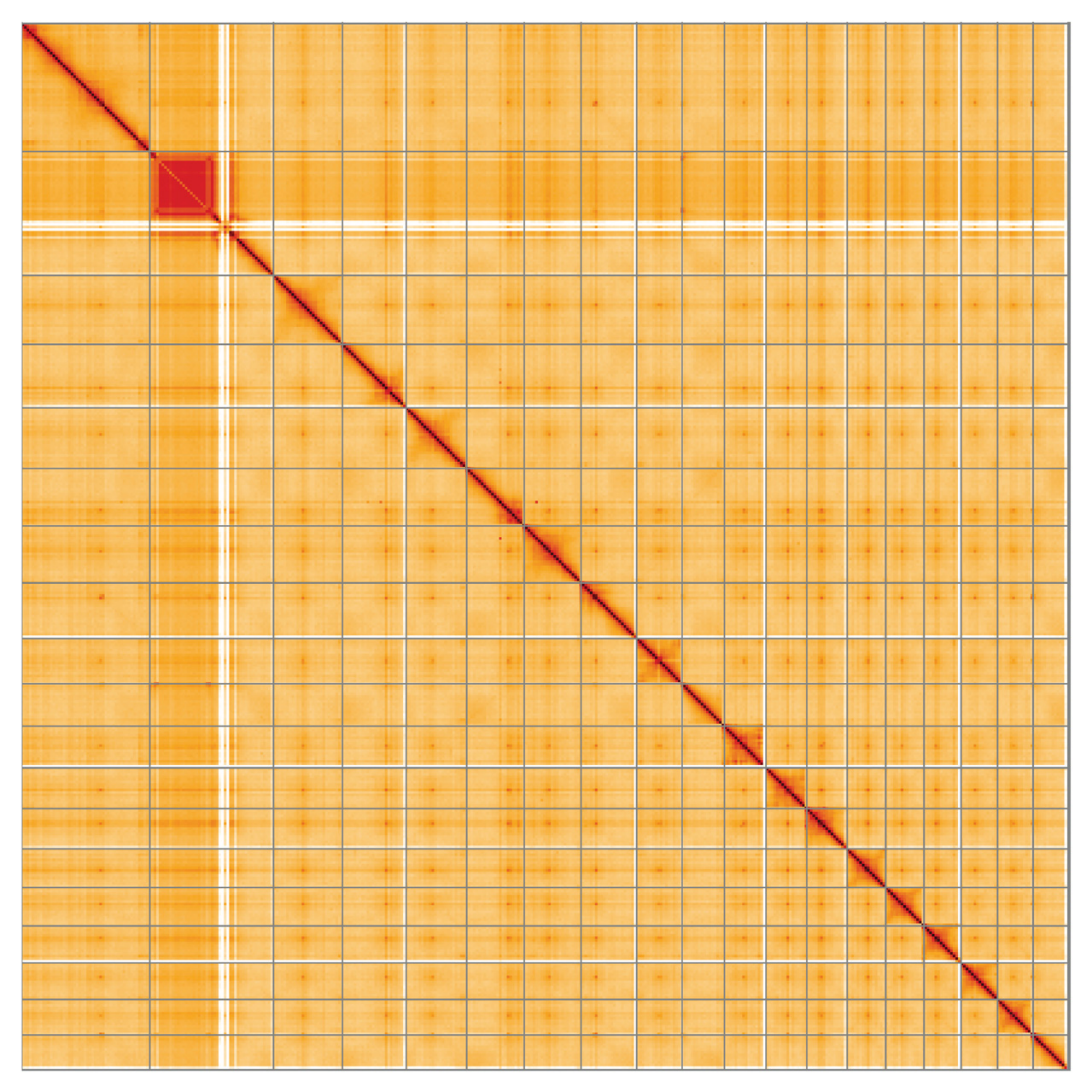
Genome assembly of *Populus nigra* subsp. *betulifolia*, ddPopNigr1.1: Hi-C contact map of the ddPopNigr1.1 assembly, visualised using HiGlass. Chromosomes are shown in order of size from left to right and top to bottom. An interactive version of this figure may be viewed at https://genome-note-higlass.tol.sanger.ac.uk/l/?d=HKCLL3icTTyCwOPRfeCJ2A.

The final assembly has a total length of 413.2 Mb in 21 sequence scaffolds with a scaffold N50 of 22.5 Mb ( Table 1). The snail plot in Figure 2 provides a summary of the assembly statistics, while the distribution of assembly scaffolds on GC proportion and coverage is shown in Figure 3. The cumulative assembly plot in Figure 4 shows curves for subsets of scaffolds assigned to different phyla. Most (99.73%) of the assembly sequence was assigned to 19 chromosomal-level scaffolds. Chromosome-scale scaffolds confirmed by the Hi-C data are named in order of size ( Figure 5; Table 2). The order and orientation of contigs along chromosome 2 between 4 Mb and 23 Mb is uncertain. While not fully phased, the assembly deposited is of one haplotype. Contigs corresponding to the second haplotype have also been deposited. The mitochondrial and plastid genomes were also assembled and can be found as contigs within the multifasta file of the genome submission.

**Table 1.** Genome data for *Populus nigra* subsp. *betulifolia*, ddPopNigr1.1.

Project accession data		
Assembly identifier	ddPopNigr1.1	
Species	*Populus nigra*	
Specimen	ddPopNigr1	
NCBI taxonomy ID	3691	
BioProject	PRJEB62046	
BioSample ID	SAMEA110450206	
Isolate information	ddPopNigr1: leaf (DNA, Hi-C and RNA sequencing)	
Assembly metrics *		*Benchmark*
Consensus quality (QV)	64.7	*≥ 50*
*k*-mer completeness	100.0%	*≥ 95%*
BUSCO **	C:97.8%[S:78.5%,D:19.3%],F:0.8%,M:1.4%,n:2,326	*C ≥ 95%*
Percentage of assembly mapped to chromosomes	99.73%	*≥ 95%*
Sex chromosomes	None	*localised homologous pairs*
Organelles	Mitochondrial assemblies: 281.85, 335.57 and 186.15 kb Plastid genome: 156.37 kb	*complete single alleles*
Raw data accessions		
PacificBiosciences SEQUEL II	ERR11458794	
Hi-C Illumina	ERR11439609	
PolyA RNA-Seq Illumina	ERR12245572	
Genome assembly		
Assembly accession	GCA_951802175.1	
*Accession of alternate haplotype*	GCA_951802205.1	
Span (Mb)	413.2	
Number of contigs	302	
Contig N50 length (Mb)	3.5	
Number of scaffolds	21	
Scaffold N50 length (Mb)	22.5	
Longest scaffold (Mb)	50.57	

* Assembly metric benchmarks are adapted from column VGP-2020 of “Table 1: Proposed standards and metrics for defining genome assembly quality” from ( Rhie *et al.*, 2021). ** BUSCO scores based on the eudicots_odb10 BUSCO set using version 5.3.2. C = complete [S = single copy, D = duplicated], F = fragmented, M = missing, n = number of orthologues in comparison. A full set of BUSCO scores is available at https://blobtoolkit.genomehubs.org/view/ddPopNigr1_1/dataset/ddPopNigr1_1/busco.

**Table 2.** Chromosomal pseudomolecules in the genome assembly of *Populus nigra* subsp. *betulifolia*, ddPopNigr1.

INSDC accession	Chromosome	Length (Mb)	GC%
OX637672.1	1	50.57	33.5
OX637673.1	2	48.86	44.5
OX637674.1	3	27.27	33.0
OX637675.1	4	25.09	34.0
OX637676.1	5	23.89	33.5
OX637677.1	6	22.73	34.5
OX637678.1	7	22.49	33.5
OX637679.1	8	21.92	34.0
OX637680.1	9	17.93	33.5
OX637681.1	10	16.71	35.5
OX637682.1	11	16.46	33.5
OX637683.1	12	16.07	33.5
OX637684.1	13	15.93	34.0
OX637685.1	14	15.21	33.5
OX637686.1	15	15.17	33.5
OX637687.1	16	14.61	33.0
OX637688.1	17	14.41	33.5
OX637689.1	18	14.01	33.5
OX637690.1	19	13.76	35.0
OX637694.1	Pltd	0.16	37.0
OX637691.1	MT1	0.28	44.5
OX637692.1	MT2	0.34	45.0
OX637693.1	MT3	0.19	45.0

The estimated Quality Value (QV) of the final assembly is 64.7 with *k*-mer completeness of 100.0%, and the assembly has a BUSCO v5.3.2 completeness of 97.8% (single = 78.5%, duplicated = 19.3%), using the eudicots_odb10 reference set ( *n* = 2,326).

Metadata for specimens, barcode results, spectra estimates, sequencing runs, contaminants and pre-curation assembly statistics are given at https://links.tol.sanger.ac.uk/species/3691.

## Methods

### Sample acquisition, genome size estimation and nucleic acid extraction

A specimen of *Populus nigra* subsp *. betulifolia* (specimen ID KDTOL10478, ToLID ddPopNigr1) was collected from along the Thames towpath in Barnes, Richmond, Surrey, UK (latitude 51.48, longitude –0.23) on 2022-05-27. The specimen was collected and identified by Maarten Christenhusz (Royal Botanic Gardens, Kew, RBG Kew) and frozen at –80 °C. The herbarium voucher associated with the sequenced plant is Christenhusz no. 9355 and is deposited in the herbarium of RBG Kew (K) (K001401000).

The genome size was estimated by flow cytometry using the fluorochrome propidium iodide and following the ‘one-step’ method as outlined in Pellicer *et al.* (2021). For this species, the General Purpose Buffer (GPB) supplemented with 3% PVP and 0.08% (v/v) beta-mercaptoethanol was used for isolation of nuclei ( Loureiro *et al.*, 2007), and the internal calibration standard was *Solanum lycopersicum* ‘Stupiké polní rané’ with an assumed 1C-value of 968 Mb ( Doležel *et al.*, 2007).

The workflow for high molecular weight (HMW) DNA extraction at the Wellcome Sanger Institute (WSI) includes a sequence of core procedures: sample preparation; sample homogenisation, DNA extraction, fragmentation, and clean-up. In sample preparation, the ddPopNigr1 sample was weighed and dissected on dry ice ( Jay *et al.*, 2023). For sample homogenisation, leaf tissue was cryogenically disrupted using the Covaris cryoPREP ^®^ Automated Dry Pulverizer ( Narváez-Gómez *et al.*, 2023). HMW DNA was extracted using the Automated Plant MagAttract v3 protocol ( Todorovic *et al.*, 2023a). HMW DNA was sheared into an average fragment size of 12–20 kb in a Megaruptor 3 system with speed setting 30 ( Todorovic *et al.*, 2023b). Sheared DNA was purified by solid-phase reversible immobilisation ( Strickland *et al.*, 2023): in brief, the method employs a 1.8X ratio of AMPure PB beads to sample to eliminate shorter fragments and concentrate the DNA. The concentration of the sheared and purified DNA was assessed using a Nanodrop spectrophotometer and Qubit Fluorometer and Qubit dsDNA High Sensitivity Assay kit. Fragment size distribution was evaluated by running the sample on the FemtoPulse system.

RNA was extracted from leaf tissue of ddPopNigr1 in the Tree of Life Laboratory at the WSI using the RNA Extraction: Automated MagMax™ *mir*Vana protocol ( do Amaral *et al.*, 2023). The RNA concentration was assessed using a Nanodrop spectrophotometer and a Qubit Fluorometer using the Qubit RNA Broad-Range Assay kit. Analysis of the integrity of the RNA was done using the Agilent RNA 6000 Pico Kit and Eukaryotic Total RNA assay.

Protocols developed by the WSI Tree of Life core laboratory are publicly available on protocols.io ( Denton *et al.*, 2023).

### Sequencing

Pacific Biosciences HiFi circular consensus DNA sequencing libraries were constructed according to the manufacturers’ instructions. Poly(A) RNA-Seq libraries were constructed using the NEB Ultra II RNA Library Prep kit. DNA and RNA sequencing was performed by the Scientific Operations core at the WSI on Pacific Biosciences SEQUEL II (HiFi) and Illumina NovaSeq 6000 (RNA-Seq) instruments. Hi-C data were also generated from leaf tissue of ddPopNigr1 using the Arima2 kit and sequenced on the Illumina NovaSeq 6000 instrument.

### Genome assembly, curation and evaluation

Assembly was carried out with Hifiasm ( Cheng *et al.*, 2021) and haplotypic duplication was identified and removed with purge_dups ( Guan *et al.*, 2020). The assembly was then scaffolded with Hi-C data ( Rao *et al.*, 2014) using YaHS ( Zhou *et al.*, 2023). The assembly was checked for contamination and corrected as described previously ( Howe *et al.*, 2021). Manual curation was performed using HiGlass ( Kerpedjiev *et al.*, 2018) and Pretext ( Harry, 2022). The organelle genomes were assembled using OATK ( Zhou, 2023).

A Hi-C map for the final assembly was produced using bwa-mem2 ( Vasimuddin *et al.*, 2019) in the Cooler file format ( Abdennur & Mirny, 2020). To assess the assembly metrics, the *k*-mer completeness and QV consensus quality values were calculated in Merqury ( Rhie *et al.*, 2020). This work was done using Nextflow ( Di Tommaso *et al.*, 2017) DSL2 pipelines “sanger-tol/readmapping” ( Surana *et al.*, 2023a) and “sanger-tol/genomenote” ( Surana *et al.*, 2023b). The genome was analysed within the BlobToolKit environment ( Challis *et al.*, 2020) and BUSCO scores ( Manni *et al.*, 2021; Simão *et al.*, 2015) were calculated.

Table 3 contains a list of relevant software tool versions and sources.

**Table 3.** Software tools: versions and sources.

Software tool	Version	Source
BlobToolKit	4.1.7	https://github.com/blobtoolkit/blobtoolkit
BUSCO	5.3.2	https://gitlab.com/ezlab/busco
Hifiasm	0.16.1-r375	https://github.com/chhylp123/hifiasm
HiGlass	1.11.6	https://github.com/higlass/higlass
Merqury	MerquryFK	https://github.com/thegenemyers/MERQURY.FK
OATK	0.1	https://github.com/c-zhou/oatk
PretextView	0.2	https://github.com/wtsi-hpag/PretextView
purge_dups	1.2.3	https://github.com/dfguan/purge_dups
sanger-tol/genomenote	v1.0	https://github.com/sanger-tol/genomenote
sanger-tol/readmapping	1.1.0	https://github.com/sanger-tol/readmapping/tree/1.1.0
YaHS	1.1a2	https://github.com/c-zhou/yahs

### Wellcome Sanger Institute – legal and governance

The materials that have contributed to this genome note have been supplied by a Darwin Tree of Life Partner. The submission of materials by a Darwin Tree of Life Partner is subject to the **‘Darwin Tree of Life Project Sampling Code of Practice’**, which can be found in full on the Darwin Tree of Life website here. By agreeing with and signing up to the Sampling Code of Practice, the Darwin Tree of Life Partner agrees they will meet the legal and ethical requirements and standards set out within this document in respect of all samples acquired for, and supplied to, the Darwin Tree of Life Project.

Further, the Wellcome Sanger Institute employs a process whereby due diligence is carried out proportionate to the nature of the materials themselves, and the circumstances under which they have been/are to be collected and provided for use. The purpose of this is to address and mitigate any potential legal and/or ethical implications of receipt and use of the materials as part of the research project, and to ensure that in doing so we align with best practice wherever possible. The overarching areas of consideration are:

•     Ethical review of provenance and sourcing of the material

•     Legality of collection, transfer and use (national and international)

Each transfer of samples is further undertaken according to a Research Collaboration Agreement or Material Transfer Agreement entered into by the Darwin Tree of Life Partner, Genome Research Limited (operating as the Wellcome Sanger Institute), and in some circumstances other Darwin Tree of Life collaborators.

## Data Availability

European Nucleotide Archive: *Populus nigra*. Accession number PRJEB62046; https://identifiers.org/ena.embl/PRJEB62046 ( Wellcome Sanger Institute, 2023). The genome sequence is released openly for reuse. The *Populus nigra* genome sequencing initiative is part of the Darwin Tree of Life (DToL) project. All raw sequence data and the assembly have been deposited in INSDC databases. The genome will be annotated using available RNA-Seq data and presented through the Ensembl pipeline at the European Bioinformatics Institute. Raw data and assembly accession identifiers are reported in Table 1.

## Author information

Members of the Royal Botanic Gardens Kew Genome Acquisition Lab are listed here: https://doi.org/10.5281/zenodo.4786680.

Members of the Plant Genome Sizing collective are listed here: https://doi.org/10.5281/zenodo.7994306.

Members of the Darwin Tree of Life Barcoding collective are listed here: https://doi.org/10.5281/zenodo.4893703.

Members of the Wellcome Sanger Institute Tree of Life Management, Samples and Laboratory team are listed here: https://doi.org/10.5281/zenodo.10066175.

Members of Wellcome Sanger Institute Scientific Operations: Sequencing Operations are listed here: https://doi.org/10.5281/zenodo.10043364.

Members of the Wellcome Sanger Institute Tree of Life Core Informatics team are listed here: https://doi.org/10.5281/zenodo.10066637.

Members of the Tree of Life Core Informatics collective are listed here: https://doi.org/10.5281/zenodo.5013541.

Members of the Darwin Tree of Life Consortium are listed here: https://doi.org/10.5281/zenodo.4783558.
