# Supplementary material for: The genome sequence of black poplar, Populus nigra subsp. betulifolia L., 1753 (Salicaceae)
Source: Wellcome Open Res. 2024 Apr 24;9:228. [Version 1] doi: 10.12688/wellcomeopenres.21300.1 (PMC11367076; doi:10.12688/wellcomeopenres.21300.1)
Supplement: Peer review history

# Reviewer report: Reviewer response for version 1

**DOI:** 10.21956/wellcomeopenres.23560.r92786

**Published:** 2024-09-01

**Authors:** Parimalan Rangan

**Affiliations:** 1 ICAR-National Bureau of Plant Genetic Resources, New Delhi, India

The present manuscript describes and reports the genome sequence of black poplar species. The species being dioecious in nature, would appreciate if the authors could generate the genome assembly for a male tree as well. 

 This will help many of the downstream analysis and understanding at genome-scale. 

 Also, authors may possibly use LAI tool to assess their assembly quality to categorize whether it is a draft or reference-scale genome. Inclusion of this metric might add value to the manuscript.

Are sufficient details of methods and materials provided to allow replication by others?

Yes

Is the rationale for creating the dataset(s) clearly described?

Yes

Are the datasets clearly presented in a useable and accessible format?

Yes

Are the protocols appropriate and is the work technically sound?

Yes

Reviewer Expertise:

Transcriptomics, genomics, molecular biology, gene cloning, plant breeding and genetics

I confirm that I have read this submission and believe that I have an appropriate level of expertise to confirm that it is of an acceptable scientific standard.

# Reviewer report: Reviewer response for version 1

**DOI:** 10.21956/wellcomeopenres.23560.r92787

**Published:** 2024-08-30

**Authors:** Marco Pessoa-Filho

**Affiliations:** 1 Brazilian Agricultural Research Corporation, Brasília, Brazil

The data note describes the genome assembly of a female individual of Populus nigra subsp. betulifolia, found in Northwestern Europe. It is an endangered species and has received attention in conservation efforts. It is a pioneer species which thrives in riparian ecosystems and provides a range of ecosystems services. Other reference genome assemblies ara available in the genus but this is the first for the species/subspecies.

 PacBio HiFi reads were obtained (54-fold coverage) and used for assembly with hifiasm. Hi-C reads generated with the Arima2 kit were used to obtain chromosome scale scaffolds with YaHS. Manual curation was carried with HiGlass and Pretext. Merqury was used to assess k-mer completeness and QV consensus quality. RNAseq data was obtained from leaf tissue, but annotation was not reported in the data note.

 Raw data and assembly were deposited in INSDC databases and are publicly available.

 The rationale for creating the dataset was clearly described. Protocols were appropriate and the work is technically sound. The datasets are clearly presented in a usable and accessible format.

**More details on genome assembly, curation and evaluation could be provided in the Materials and Methods and would enrich the data note and allow its replication:**

 1) Was Hifiasm run with default parameters?

 2) Considering that Hi-C data was available, was it used as input to Hifiasm for phasing?

 3) What output was further used in the pipeline for curation and scaffolding? Was it the p_ctg? The hap1_ctg?

 4) Considering that hifiasm already includes purging of haplotype duplications, why was purge_dups used? Was hifiasm run without haplotype duplication removal?

 5) What dataset was used as input to Merqury along with the assembly to assess k-mer completeness and QV consensus quality values?

 6) Table 3 lists MerquryFK, not the original Merqury. This should be mentioned in the text.

 7) Authors should describe what exactly the pipelines sanger-tol/readmapping and sanger-tol/genomenote do.

**Additional comments:**

 Background, third paragraph:"...after which is soon became popular" should be "after which it soon became popular"

Are sufficient details of methods and materials provided to allow replication by others?

Partly

Is the rationale for creating the dataset(s) clearly described?

Yes

Are the datasets clearly presented in a useable and accessible format?

Yes

Are the protocols appropriate and is the work technically sound?

Yes

Reviewer Expertise:

Plant genomics, bioinformatics

I confirm that I have read this submission and believe that I have an appropriate level of expertise to confirm that it is of an acceptable scientific standard, however I have significant reservations, as outlined above.

# Reviewer report: Reviewer response for version 1

**DOI:** 10.21956/wellcomeopenres.23560.r88962

**Published:** 2024-07-26

**Authors:** Boas Pucker

**Affiliations:** 1 Technische Universitat Braunschweig, Brunswick, Lower Saxony, Germany

This genome announcement by Christenhusz and Bastaiaanse describes the genome sequence of Populus nigra. The project was done by the Darwin Tree of Life Consortium. The genome sequence is certainly a helpful resources and it is good to see all data released.

 Here are some comments to improve the manuscript:

- "The genome sequence is 413.2 megabases in span." ...this sentence should be rephrased.
- chromosomal pseudomolecules > pseudochromosomes
- "chondrome" and "plastome" should be used to refer to these subgenomes.
- "This resilient species thrives in riparian ecosystems." ... what is the evidence for this broad statement?
- "Such forests are not only biodiversity hotspots,"... It appears that the cited reference Van Looy et al., 2013 does not conclude (and also not study in detail) that Populus nigra forest are a biodiversity hotspot.
- "Regrettably," ... Please rephrase, because this reads like activism and not like objective science.
- There are some redundant sentences in the introduction. For example, ecosystem services are reported twice.
- which is soon > which it soon
- present the first genome > present the first genome sequence
- "The reference genome will enable the systematic assessment and characterization of sequence variation among natural populations of black poplar, without the need to rely on reference-guided mapping and variant calling based solely on the reference genome of P. trichocarpa Torr. & A.Gray ex. Hook." ... This sentence seems to contradict itself: a reference genome sequence is generated to avoid analyzing based on a reference. If the intended meaning is that the current reference genome sequence should be replaced the sentence should be shortened.
- "Single Nucleotide Polymorphisms (SNPs)" .... I would recommend to use the term Single Nucleotide Variants (SNVs) instead, because it does not make any implications about the functional consequences of sequence differences. SNPs would not have a detrimental impact by definition (polymorphism).
-  Presenting just one project to demonstrate the usefulness of the genome sequence is substantially narrowing down and reducing the importance. It generates the impression that only this project needs the genome sequence and after 2025 it is not relevant anymore. I would suggest to remove this statement and to propose broader applications.
- "Manual assembly curation corrected 156 missing joins or mis-joins and removed 11 haplotypic duplications," ... These steps would not be reproducible. Please explain in detail how this was done.
- "subsets of scaffolds assigned to different phyla" ... What are these different phyla? This also needs to be explained in the legend of Figure 4.
- The specimen was frozen at -80°C? Please explain why this was done.
- The DNA extraction and clean-up workflow needs to be described.
- "Protocols developed by the WSI Tree of Life core laboratory are publicly available on protocols.io (Denton et al., 2023)." .... It is not clear to me, to which section this sentence belongs and which protocols are mentioned here.
- The sequencing should be described with more technical details. For example, how many PCR cycles in the Illumina library prep? Which amount of nucleic acids was used for each of the sequencing attempts?
- The assembly process lacks version numbers for the mentioned tools and also the necessary details to repeat all steps. Please include all parameters used.
- Content off Figure 3 and Figure 4 could be summarized in the text to make the manuscript more concise.

Are sufficient details of methods and materials provided to allow replication by others?

Partly

Is the rationale for creating the dataset(s) clearly described?

Partly

Are the datasets clearly presented in a useable and accessible format?

Yes

Are the protocols appropriate and is the work technically sound?

Partly

Reviewer Expertise:

plant genomics, specialized plant metabolism, applied bioinformatics

I confirm that I have read this submission and believe that I have an appropriate level of expertise to confirm that it is of an acceptable scientific standard, however I have significant reservations, as outlined above.
